# Haploid Genome Analysis Reveals a Tandem Cluster of Four *HSP20* Genes Involved in the High-Temperature Adaptation of *Coriolopsis trogii*

**DOI:** 10.1128/spectrum.00287-21

**Published:** 2021-08-18

**Authors:** Lining Wang, Baosheng Liao, Lu Gong, Shuiming Xiao, Zhihai Huang

**Affiliations:** a Institute of Bioengineering, Guangdong Academy of Sciences, Guangzhou, People’s Republic of China; b Key Laboratory of Quality Evaluation of Chinese Medicine of the Guangdong Provincial Medical Products Administration, the Second Clinical College, Guangzhou University of Chinese Medicine, Guangzhou, People’s Republic of China; c Institute of Chinese Materia Medica, China Academy of Chinese Medical Sciences, Beijing, People’s Republic of China; University of Molise

**Keywords:** haplotype genome, *HSP20*, thermotolerance, *Coriolopsis trogii*

## Abstract

Coriolopsis trogii is a typical thermotolerant basidiomycete fungus, but its thermotolerance mechanisms are currently unknown. In this study, two monokaryons of C. trogii strain Ct001 were assembled: Ct001_29 had a genome assembly size of 38.85 Mb and encoded 13,113 genes, while Ct001_31 was 40.19 Mb in length and encoded 13,309 genes. Comparative intra- and interstrain genomic analysis revealed the rich genetic diversity of *C. trogii*, which included more than 315,194 single-nucleotide polymorphisms (SNPs), 30,387 insertion/deletions (indels), and 1,460 structural variations. Gene family analysis showed that the expanded families of *C. trogii* were functionally enriched in lignocellulose degradation activities. Furthermore, a total of 14 allelic pairs of heat shock protein 20 (*HSP20*) genes were identified in the *C. trogii* genome. The expression profile obtained from RNA sequencing (RNA-Seq) showed that four tandem-duplicated allelic pairs, *HSP20.5* to *HSP20.8*, had more than 5-fold higher expression at 35°C than at 25°C. In particular, *HSP20.5* and *HSP20.8* were the most highly expressed *HSP20* genes. Allelic expression bias was found for *HSP20.5* and *HSP20.8*; the expression of *Ct29HSP20.8* was at least 1.34-fold higher than that of *Ct31HSP20.8*, and that of *Ct31HSP20.5* was at least 1.5-fold higher than that of *Ct29HSP20.5*. The unique structural and expression profiles of the *HSP20* genes revealed by these haplotype-resolved genomes provide insight into the molecular mechanisms of high-temperature adaptation in *C. trogii*.

**IMPORTANCE** Heat stress is one of the most frequently encountered environmental stresses for most mushroom-forming fungi. Currently available fungal genomes are mostly haploid because high heterozygosity hinders diploid genome assembly. Here, two haplotype genomes of *C. trogii*, a thermotolerant basidiomycete, were assembled separately. A conserved tandem cluster of four *HSP20* genes showing allele-specific expression was found to be closely related to high-temperature adaptation in *C. trogii*. The obtained haploid genomes and their comparison offer a more thorough understanding of the genetic background of *C. trogii*. In addition, the responses of *HSP20* genes at 35°C, which may contribute to the growth and survival of *C. trogii* at high temperatures, could inform the selection and breeding of elite strains in the future.

## INTRODUCTION

Coriolopsis trogii, previously known as Trametes trogii (https://www.mycoguide.com/guide/fungi/basi/agar/poly/poly/trma/trogii), is a globally distributed white-rot basidiomycete that has been recognized as an excellent source of ligninolytic enzymes and thermostable laccases (1–6). The crude extract and exopolysaccharides of *C. trogii* also show strong pharmacological effects, such as anticancer and antioxidative effects (7–9). Most mushroom-forming fungi are sensitive to temperature, and temperatures above 25°C during the cultivation process may seriously affect the yield and quality of the fruiting bodies (10, 11). The optimal temperature for mycelial growth in *C. trogii* is about 35°C, which is significantly higher than that for most filamentous fungi (5), making it a good candidate for studying the heat resistance or adaptation of fungi.

Heat shock protein 20 (*HSP20*) genes, which encode a fascinating group of molecular chaperones and comprise a major family of *HSP* genes, possess a conserved sequence of 80 to 100 amino acid residues, called an α-crystallin domain (ACD), in their C-terminal region (12). HSP20s bind to partially folded or denatured proteins to prevent proteins from irreversible aggregation and keep them stable (13). *HSP20* genes are a major family of *HSP* genes induced by elevated temperature that are associated with stress responses in a range of different species (12, 14–19). In addition to environmental adaption, *HSP20* genes are associated with development in plants (12, 20, 21) and bacteria (22). However, the gene number and structure of the *HSP20* family and their relationship with thermotolerance in *C. trogii* are currently unknown.

In basidiomycetes, two different nuclei coexist side by side in one cell during the majority of their life cycle (23). To achieve better assembly and annotation results, monokaryon strains derived from protoplasts or single spores are usually selected to conduct genome sequencing (24–27). However, considerable phenotypic and genetic diversity is found between different monokaryons, strains, or haplotypes (23, 28, 29), and thus more genomes may provide a more comprehensive genetic background of a single species. Currently, only one whole-genome sequence for a monokaryon of *C. trogii* (S0301) is available (30). Additional genomic resources are thus critically important for realizing a more comprehensive and in-depth understanding of *C. trogii*.

In our current study, two protoplast-derived mating-compatible monokaryons, namely, Ct001_29 (mating type A_x_B_x_) and Ct001_31 (mating type A_y_B_y_), of *C. trogii* strain Ct001 were sequenced and assembled. Comparative genomic analysis was conducted, and genetic variations were identified. Through comprehensive RNA sequencing (RNA-Seq) analysis and allele-specific expression analysis (ASE), genes encoding HSP20s, especially the duplicated allelic pairs *Ct29HSP20.8/Ct31HSP20.8* and *Ct29HSP20.5/Ct31HSP20.5*, were suggested to be the major *HSP* genes participating in the high-temperature adaptation of *C. trogii*.

## RESULTS

### Assembly and annotation of the Ct001_29 and Ct001_31 genomes.

**FIG 1:**
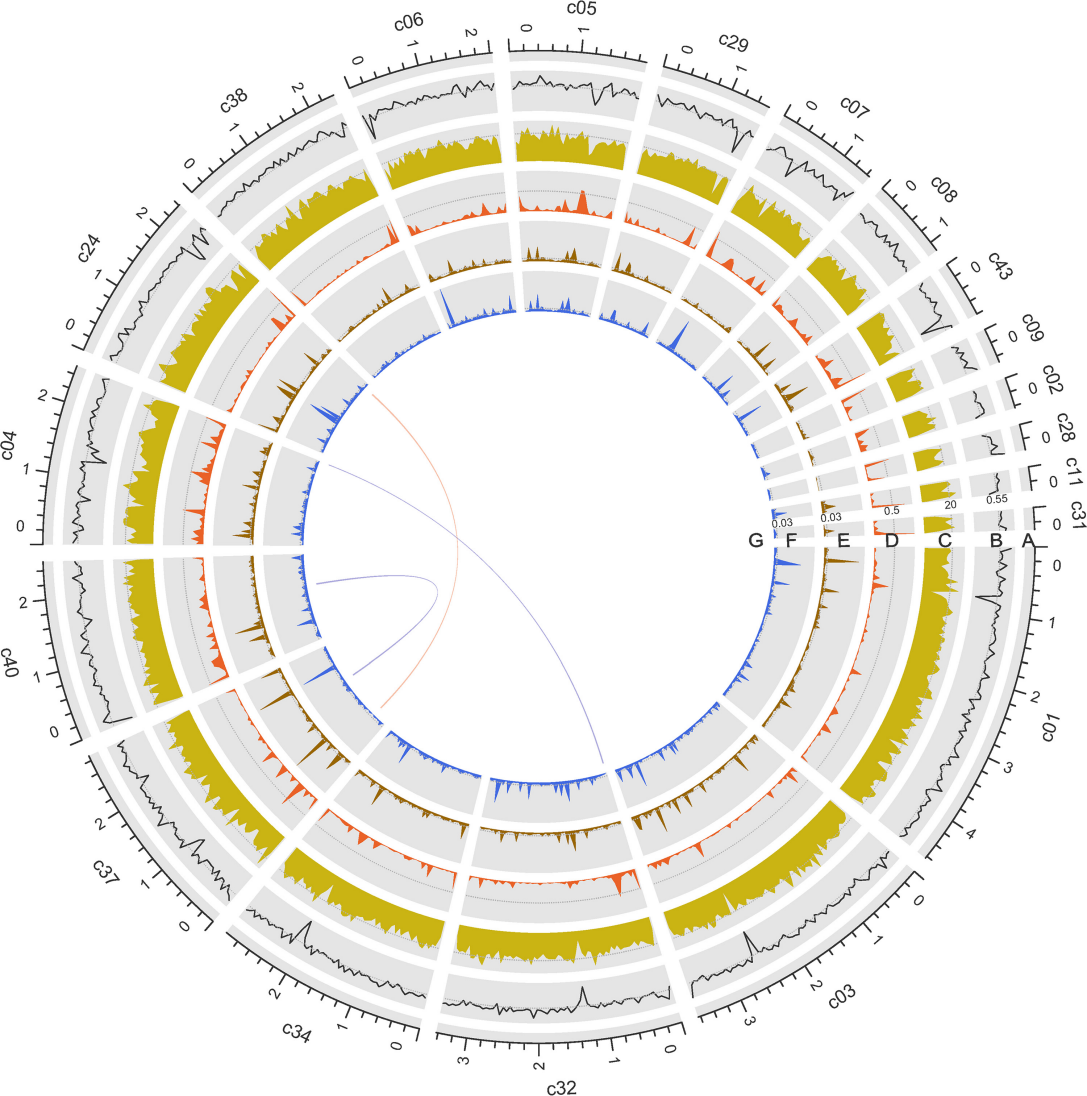
Global view of the Ct001_29 genome. (A) The longest 20 contigs of Ct001_29; (B) G+C percentage; (C) gene density; (D) repeat content; (E) variant density in Ct001_29 versus that in Ct001_31; (F) variant density in Ct001_29 versus that in S0301 (30); (G) syntenic blocks (sequence length, ≥5 kb) within the Ct001_29 genome. All of the statistics were calculated over 50-kb nonoverlapping windows.

The diploid strain Ct001 was isolated from the field in Shandong, China, in 2019. A total of 60 monokaryons were obtained, of which two mating-compatible haploids were randomly selected (Ct001_29 and Ct001_31) for this study. In total, 100× to 120× coverage from Illumina reads and 50× to 60× coverage from PacBio reads was generated for each genome. The 38.85-Mb assembly of Ct001_29 consisted of 43 contigs, with a contig *N*_50_ of 2.53 Mb, and the 40.19-Mb assembly of Ct001_31 consisted of 38 contigs, with a contig *N*_50_ of 2.48 Mb (Table 1 and Fig. 1A). The high degree of completeness of these assemblies was supported by the *k*-mer spectrum (see Fig. S1 in the supplemental material) and by Benchmarking Universal Single-Copy Ortholog (BUSCO) analysis. A total of 283 BUSCOs (97.5% of genes in the fungal lineage of odb9) were identified in both assemblies, of which 282 were complete and only one was a fragment. These two genomes had comparable assembly quality with that of a previously published *C. trogii* draft genome (S0301) (30) in total genome size and *N*_50_ value (Table 1).

**TABLE 1:** Statistics for assembly features of *C. trogii* nuclear genomes

Statistic	*C. trogii* genome assembly		
	Ct001_29	Ct001_31	S0301^*a*^
No. of contigs	43	38	29
Total length (Mb)	38.85	40.19	39.88
Longest contig (Mb)	4.63	4.78	4.82
Shortest contig (kb)	1.52	1.92	20.37
Avg contig length (kb)	903.50	1,057.75	1,375.01
Contig *N*_50_ (Mb)	2.53	2.48	2.4
GC content (%)	55.40	55.37	55.47
Repeat content (%)	7.64	10.20	
No. of protein-coding genes	13,113	13,309	14,508

a The S0301 genome sequence was previously published by Liu et al. (30).

Repetitive sequences represented 7.97% and 10.18% of Ct001_29 and Ct001_31, respectively (see Table S1 in the supplemental material). Long terminal repeats (LTR) were dominant repeat elements in both genomes, with Ct001_29 having a percentage of 2.34% and Ct001_31 having a percentage of 3.45%. A total of 13,113 protein-coding genes were predicted in Ct001_29, while 13,309 were predicted in Ct001_31. Low-GC-content regions tended to have low gene density (Fig. 1B and C). Gene functional annotation of Ct001_29 revealed that approximately 86.97% (11,402), 58.48% (7,667), and 77.58% (10,171) of the annotations could be assigned using data from InterProScan, Pfam, and UniProt, respectively.

### Evolution of the *C. trogii* genome.

**FIG 2:**
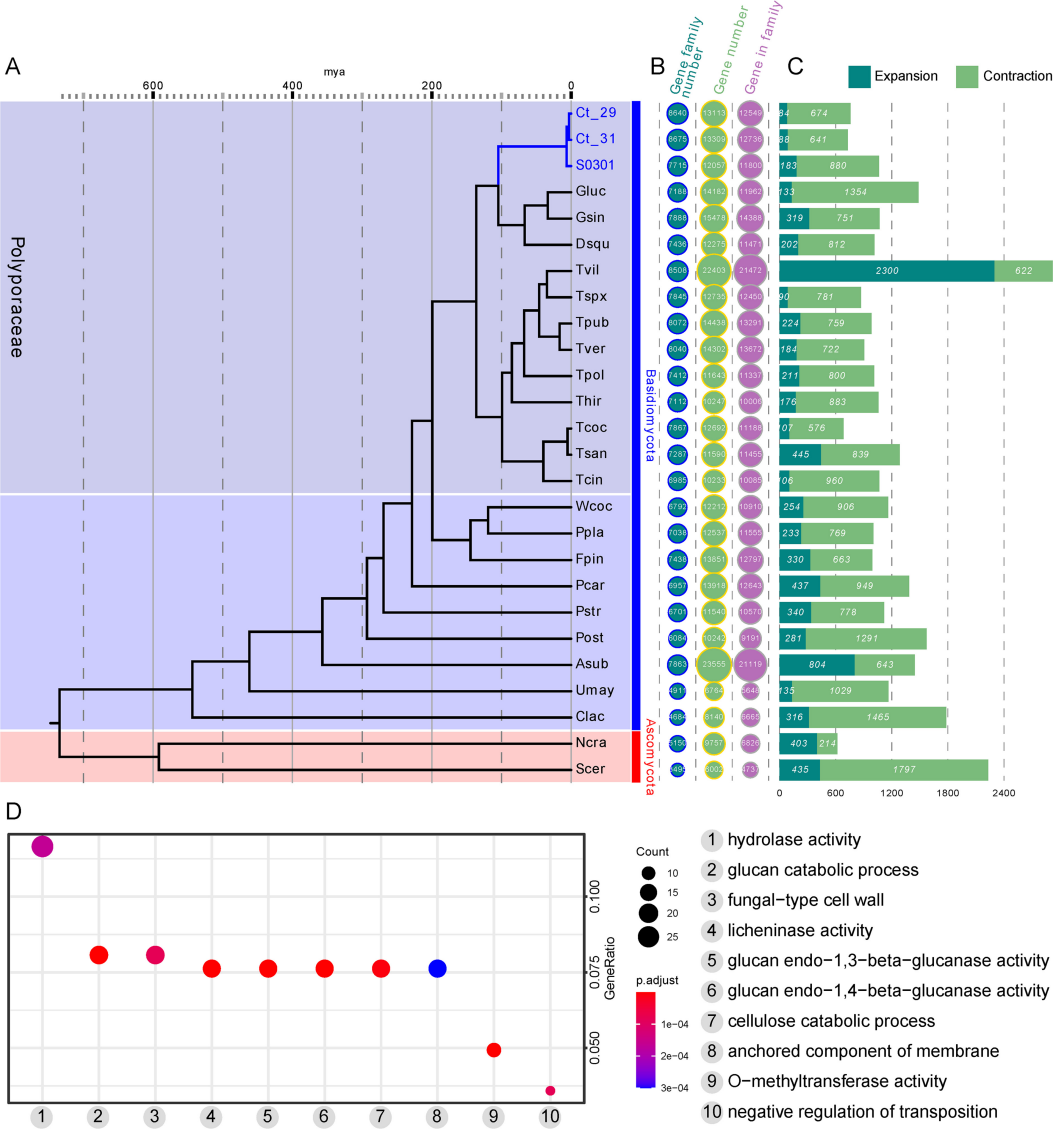
Phylogenetic analysis of *C. trogii*. (A) Phylogenetic tree (maximum likelihood) constructed based on 176 single-copy genes in *C. trogii* and 23 other species using RAxML. Mya, million years ago. The bootstrap values for the most recent common ancestor (MRCA) were 65% for Umay versus Clac, 76% for Pstr versus Pcar, and 100% for others. Gluc, *Ganoderma lucidum*; Gsin, *Ganoderma sinense*; Dsqu, *Dichomitus squalens*; Tvil, *Trametes villosa*; Tspx, *Trametes sp. AH28-2*; Tpub, *Trametes pubescens*; Tver, *Trametes versicolor*; Tpol, *Trametes polyzona*; Thir, *Trametes hirsute*; Tcoc, *Trametes coccinea*; Tsan, *Trametes sanguinea*; Tcin, *Trametes cinnabarina*; Wcoc, *Wolfiporia cocos*; Ppla, *Postia placenta*; Fpin, *Fomitopsis pinicola*; Pcar, *Phanerochaete carnosa*; Pstr, *Puccinia striiformis*; Post, *Pleurotus ostreatus*; Asub, *Auricularia subglabra*; Umay, *Ustilago maydis*; Clac, *Cystobasidiopsis lactophilus*; Ncra, *Neurospora crassa*; Scer, *Saccharomyces cerevisiae*, and S0301, *C. trogii*. (B) Gene families identified in 24 species. Gene family number represents the number of gene families identified in the corresponding species; the gene number represents the number of all of the predicted genes in the genome of the corresponding species; “gene in family” represents the total number of genes that can be classified into gene families. (C) Gene family expansion and contraction. Ct_29 and Ct_31 represent Ct001_29 and Ct001_31, respectively. (D) Functional enrichment of the expanded genes.

To investigate the evolutionary history of the *C. trogii* genome, an orthologous gene analysis using *C. trogii* and 23 other representative species (see Table S2 in the supplemental material) was conducted. A total of 176 single-copy orthologous genes were used for phylogenetic tree construction. The phylogenetic tree showed that the estimated divergence time between the *C. trogii* lineage and the Ganoderma lucidum and Ganoderma sinensis lineages was around 104 million years ago (Mya) (Fig. 2A). There were 84, 88, and 168 gene families that had possibly expanded in the Ct001_29, Ct001_31, and S0301 (30) strains, respectively, while 674, 641, and 880 gene families, respectively, appeared to have contracted compared to those in other taxa in this tree (Fig. 2B and C). Genes belonging to glycoside hydrolase families, especially *GH16*, were significantly enriched in the observed expanded genes (Fig. 2D), which may be related to the strong lignocellulose degradation ability of this species.

### Comparative genomics among *C. trogii* strains.

**FIG 3:**
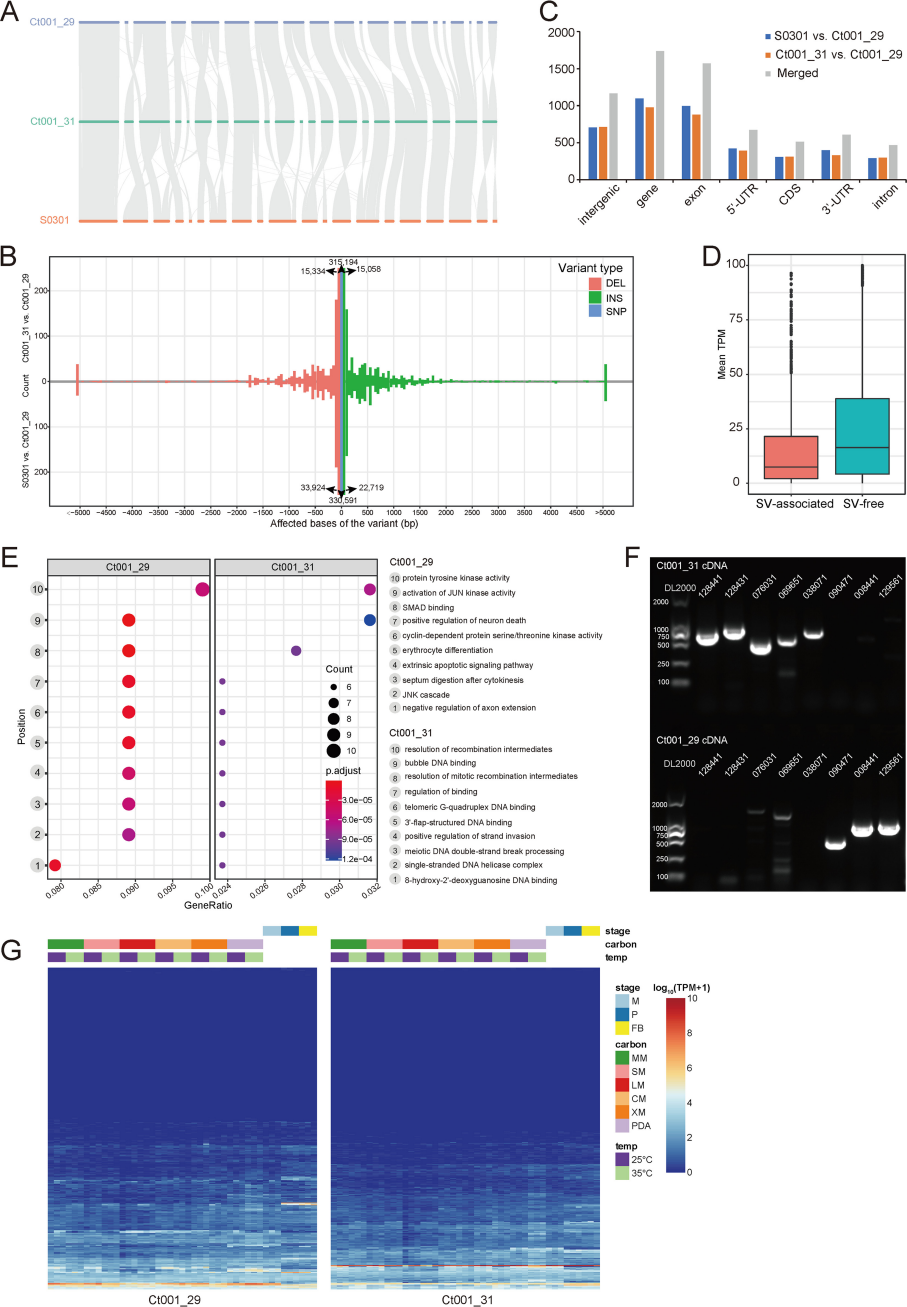
Comparative analysis of *C. trogii* genomes. (A) Sequence syntenic alignment of S0301 (30), Ct001_31, and Ct001_29. Gray lines indicate shared syntenic blocks; horizontal lines indicate contigs of Ct001_29 (blue), Ct001_31 (green), and S0301 (orange). (B) Variant length distribution of S0301 versus that of Ct001_29 and that of Ct001_31 versus that of Ct001_29. The affected base size of a single-nucleotide polymorphism (SNP) (blue bar) is 0, the affected base size of an insertion (green bar) is >0, and the affected base size of a deletion (pink bar) is <0. DEL, deletion; INS, insertion. (Upper) Variant numbers of the comparison between Ct001_29 and Ct001_31; (lower) variant numbers of the comparison between Ct001_29 and S0301. (C) Number of structural variations (SVs) overlapping with specific genomic features. The “merged” columns represent numbers of SVs from the union of two comparisons. (D) Expression of SV-associated genes and SV-free genes. TPM, transcripts per million. (E) Functional enrichment of Ct001_29-specific genes and Ct001_31-specific genes. (F) Agarose gel electrophoresis of haplotype-specific genes. Genes of 128441, 128431, 076031, 069651, and 038071 are Ct001_31 specific, whereas the others are Ct001_29 specific. (G) Expression heatmap of haplotype-specific genes under different conditions. Carbon, carbon source; M, mycelium; P, primordium; FB, fruiting body; MM, glucose; SM, sucrose; LM, lignin; CM, cellulose; XM, xylan; PDA, potato dextrose agar.

To capture the genomic diversity of *C. trogii* in terms of the sequences from both interstrain (S0301 versus Ct001_29) and intrastrain (Ct001_31 versus Ct001_29) comparisons, the sequences of Ct001_29, Ct001_31, and S0301 (30) were compared in a pairwise manner. Overall, strong syntenic relationships between these genomes were observed, while several structural differences existed (Fig. 3A). Substantial variations were identified, including 330,591 single-nucleotide polymorphisms (SNPs), 56,638 insertion/deletions (indels), and 1,563 structural variations (SVs), in the interstrain comparisons, as well as 315,194 SNPs, 30,387 indels, and 1,460 SVs in the intrastrain comparisons (Fig. 3B). The variants were distributed unevenly across the whole genome, with some regions possessing high variant density (Fig. 1E and F). We found that most of these SVs had lengths of less than 2 kb. Among the interstrain SVs, 704 were distributed in intergenic regions and 1,097 were distributed in gene regions, with most (997) occurring in exons. Consistently, among the intrastrain SVs, 714 were distributed in intergenic regions and 975 were distributed in gene regions, with most (878) occurring in exons (Fig. 3C). Randomly selected SNPs and indel loci (except primer indel_5) were then amplified successfully, and these loci were confirmed by Sanger sequencing (Fig. S2A to C). A set of randomly selected SVs was confirmed by sequence amplification and agarose gel electrophoresis (see Fig. S2D in the supplemental material). On average, SV-associated genes in Ct001_29 showed relatively lower expression than that of SV-free genes (Fig. 3D), which indicated that SVs may influence the expression of nearby genes.

In the genome comparison between S0301 and Ct001_29 (interstrain), 2,236 genes were highly conserved (variable bases accounted for less than 0.2% of a gene) and 3,257 were highly variable (variable bases accounted for more than 2% of a gene). In the genome comparison between Ct001_31 and Ct001_29 (intrastrain), 2,822 genes were highly conserved and 2,643 were highly variable. The conserved genes were functionally enriched in genome stability maintenance, such as DNA recombination, endonuclease activity, and DNA integration. The variable genes detected, however, were functionally enriched in catalyzing activities, such as oxidoreductase and monooxygenase.

In total, Ct001_31 had 365 haplotype-specific genes, and Ct001_29 had 360 haplotype-specific genes (the list of all of the haplotype-specific genes can be obtained at http://www.gpgenome.com:8080/species/62343). Ct001_31-specific genes were enriched in DNA-binding functions, and Ct001_29-specific genes were enriched in signal transduction-related functions (Fig. 3E). To validate the authenticity of the haplotype-specific genes, we used complementary DNA (cDNA) from Ct001_29 and Ct001_31 as the templates for PCR amplifications. Based on the band patterns observed (existence or absence) by agarose gel electrophoresis, five selected Ct001_31-specific genes and three Ct001_29-specific genes were further validated (Fig. 3F). Most haplotype-specific genes showed low expression levels under all of the culture conditions (Fig. 3G), and about half of them even exhibited no expression (43.06% of Ct001_29-specific genes and 47.40% of Ct001_31-specific genes), indicating their accessary roles in the growth and development of *C. trogii*.

### Expression profile of *C. trogii* under different conditions.

**FIG 4:**
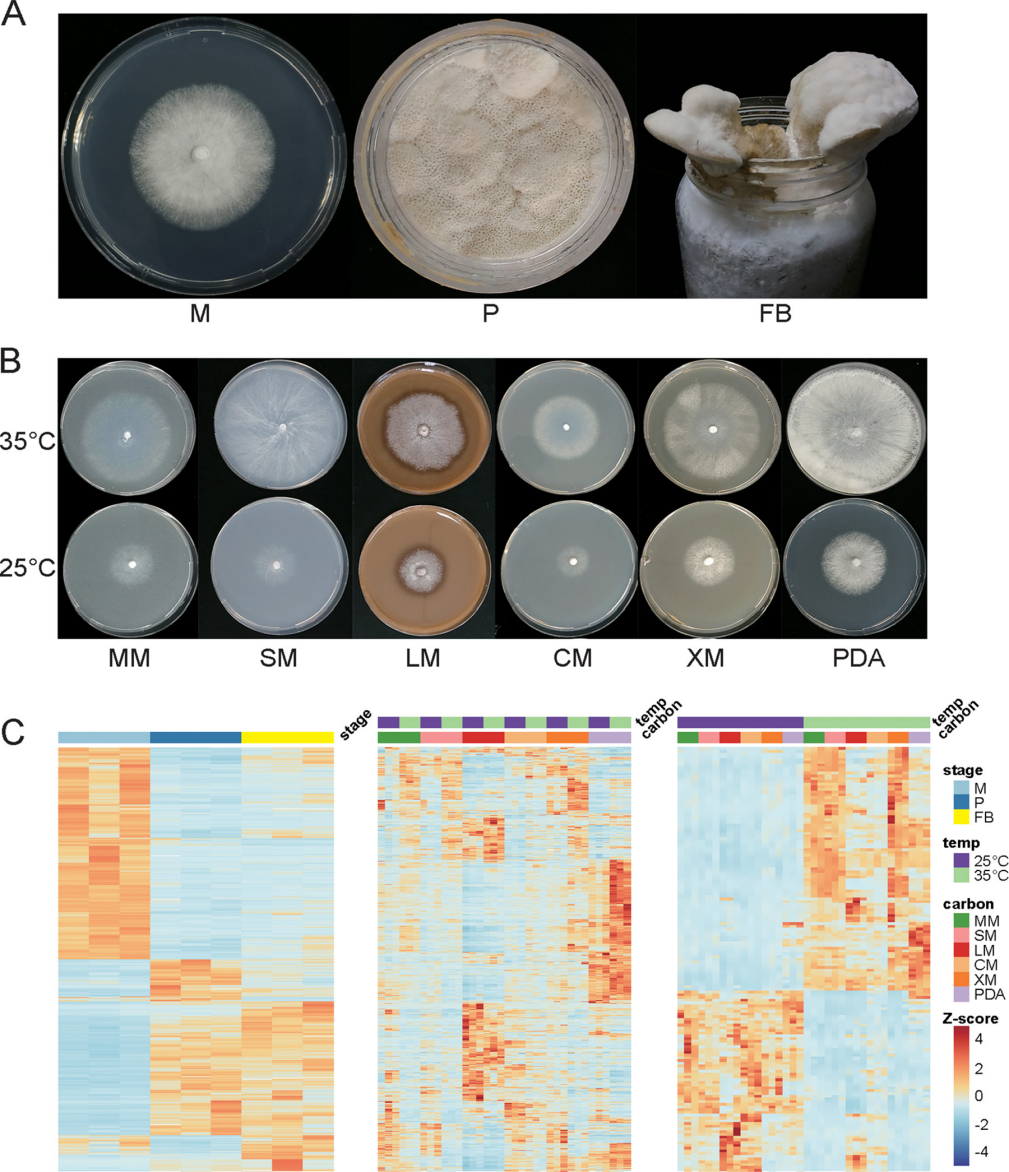
Expression profile of *C. trogii* diploid strain Ct001 under different conditions. (A) Different developmental stages of *C. trogii*. M, mycelium; P, primordium; FB, fruiting body. (B) Growth status of mycelia cultured on different carbon sources and at different temperatures. MM, glucose; SM, sucrose; LM, lignin; CM, cellulose; XM, xylan; PDA, potato dextrose agar. (C) Heatmap of differentially expressed genes under different conditions. “Carbon” indicates the carbon source. The Z-score is the standardized value of transcripts per million for each gene.

Ct001 has a wide spectrum of carbon sources that it can use (carbon utilization ability), and it can produce fruiting bodies on a variety of natural substrates (see Fig. S3A in the supplemental material). The optimal growth rate of Ct001 at 35°C compared to that at 25°C under all of the tested conditions confirmed the high-temperature adaptation ability of this organism (Fig. S3B and C). To investigate the molecular mechanisms of high-temperature adaptation in *C. trogii*, RNA-Seq analysis of mycelia cultured on different carbon sources and different temperatures, as well as during the different developmental stages of Ct001, was conducted (Fig. 4A and B).

Specific expression was observed under different conditions (developmental stages, carbon sources, or temperatures) (Fig. 4C). During different developmental stages, 2,628 differentially expressed genes (DEGs) (vegetative mycelia versus primordia) and 430 DEGs (primordia versus fruiting bodies) were identified (see Fig. S4C in the supplemental material), and these developmental DEGs were enriched in hydrolase activity, polysaccharide catabolic process, and aromatic compound catabolic process. A total of 2,243 carbon-related DEGs were identified (Fig. S4A), and these genes were enriched in functions of heme binding, monooxygenase activity, and the polysaccharide/aromatic compound/glucan/cellulose catabolic process. A total of 45 overlapping temperature-related DEGs were identified in growth on all six carbon groups, while this number was 155 under any combination of five different carbon groups (Fig. S4B); these 155 DEGs were enriched in chaperone complex and cellular response to unfolded/misfolded proteins. Notably, four *HSP20* genes (*HSP20.5* to *HSP20.8*) were differentially expressed under all of the tested conditions and showed extremely high expression levels at 35°C.

### The *HSP20* gene family is specifically involved in the high-temperature adaptation of *C. trogii*.

**FIG 5:**
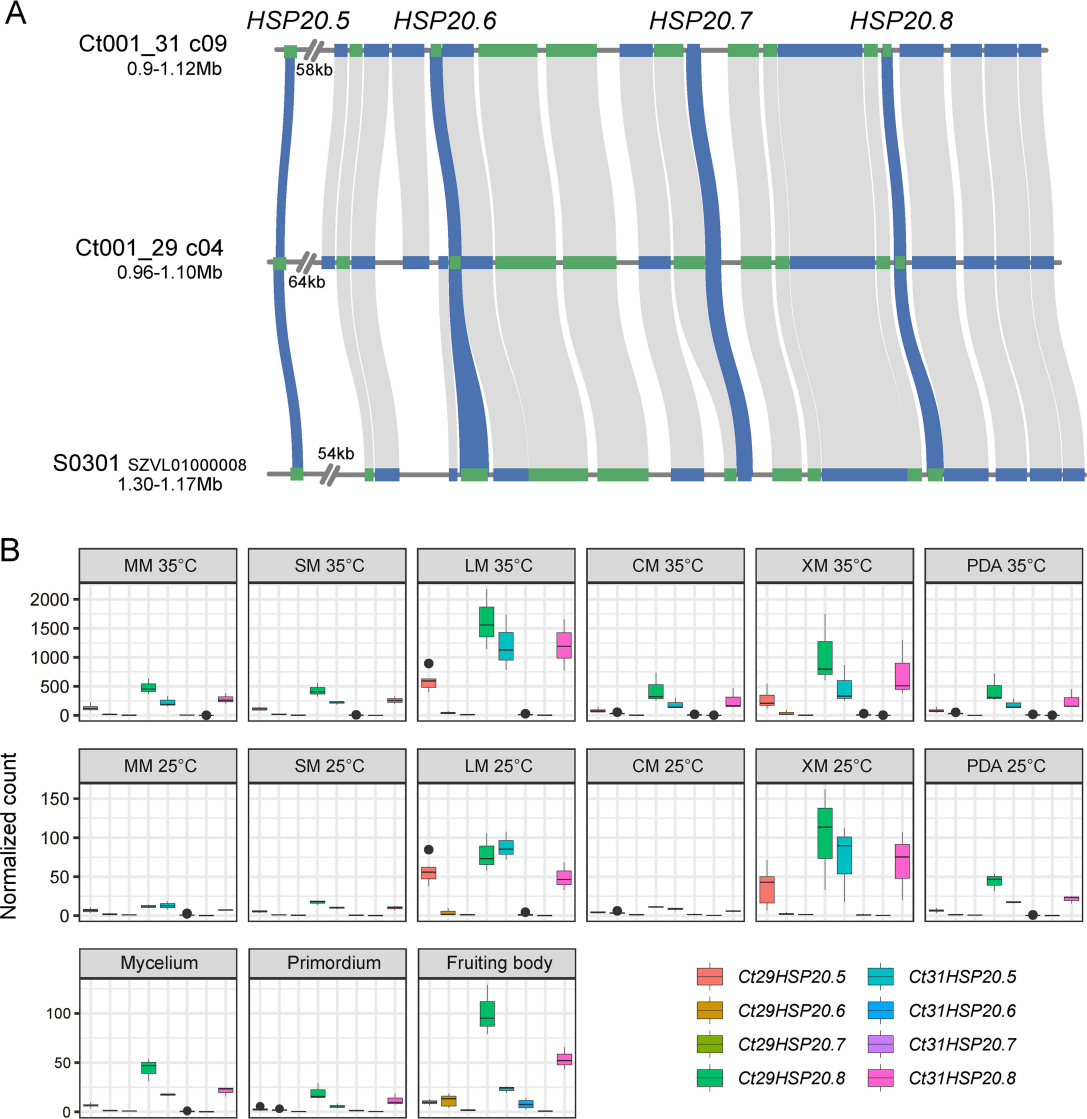
Structure and expression profile of the *C. trogii HSP20* tandem cluster. (A) Syntenic relationship of genes in the *HSP20* tandem cluster of Ct001_29, Ct001_31, and S0301 (30). Green blocks represent genes on a forward strand, while blue blocks represent genes on a reverse strand. Wavy lines indicate shared syntenic genes, and blue wavy lines indicate syntenic relationship of *HSP20* genes. (B) Normalized read counts of *HSP20* genes under different conditions. MM, glucose; SM, sucrose; LM, lignin; CM, cellulose; XM, xylan.

In total, 14 allelic pairs of *HSP20* genes were identified in *C. trogii* (see Fig. S5 in the supplemental material), among which 8 pairs belonging to 3 groups were duplicated. Specifically, the group involving *HSP20.5* to *HSP20.8* was tandem duplicated, forming a tandem cluster (Fig. 5A). According to our RNA-Seq results, most of the *HSP20* genes were more highly expressed at 35°C than at 25°C (Fig. S5C). The expression of *HSP20.5* to *HSP20.8* was then validated using quantitative PCR (qPCR). Due to their high sequence similarity, only one primer pair was designed and was used to detect their total expression (that of *Ct29HSP20.5* to *Ct29HSP20.8* and *Ct31HSP20.5* to *Ct31HSP20.8*) as a whole. The expression levels at 35°C were all significantly higher (larger than 5-fold change) than those at 25°C under all of the tested conditions (see Fig. S6A in the supplemental material), indicating that these four *HSP20* genes were widely involved in the high-temperature adaptation of *C. trogii*.

In this cluster, the allele pairs *Ct29HSP20.5*-*Ct31HSP20.5* and *Ct29HSP20.8*-*Ct31HSP20.8* showed high expression, while *Ct29HSP20.6*-*Ct31HSP20.6* and *Ct29HSP20.7*-*Ct31HSP20.7* showed quite low expression under all of the tested conditions at the mycelia stage. Similarly, *Ct29HSP20.8*-*Ct31HSP20.8* showed high expression across the developmental stages (Fig. 5B). According to our sequence similarity analysis results, the *Ct29HSP20.5*-*Ct31HSP20.5* and *Ct29HSP20.8*-*Ct31HSP20.8* allelic pair genes had similar promoter regions (Fig. S6B), indicating that they may be regulated by similar *cis*-regulation. Allele dominance was found in *Ct29HSP20.5*-*Ct31HSP20.5* and *Ct29HSP20.8*-*Ct31HSP20.8*, as the expression of *Ct31HSP20.5* was consistently higher (at least 1.5-fold) than that of *Ct29HSP20.5*, while that of *Ct29HSP20.8* was consistently higher (at least 1.3-fold) than that of *Ct31HSP20.8* (Fig. 5B). To validate the high expression of *Ct29HSP20.5*-*Ct31HSP20.5* and *Ct29HSP20.8*-*Ct31HSP20.8*, we amplified a 563-bp sequence from this cluster and conducted clone sequencing. Among the 56 tested clones, the proportion of *Ct29HSP20.5*:*Ct31HSP20.5*:*Ct29HSP20.6*:*Ct31HSP20.6*:*Ct29HSP20.7*:*Ct31HSP20.7*:*Ct29HSP20.8*:*Ct31HSP20.8* was 7:19:1:0:0:0:20:9 (Fig. S6C and D).

## DISCUSSION

“Pangenome” and comparative genomics studies based on large numbers of high-quality genomes have become trendy research approaches as of late, especially in model fungal species, such as Saccharomyces cerevisiae (31) and Aspergillus spp. (32, 33). These studies have revealed the abundant genetic diversity in a given species and provide a solid foundation for the resolution of genotype-phenotype relationships, as well as illustrating the importance of having more complete genomes in a population. Several macrofungal taxa, such as G. lucidum, have more than one set of genomes available, and these genomes are typically from different strains with different phenotypes (26, 34). However, genome information from different karyotypes of the same strain is still limited. In this study, we successfully assembled two haplotype genomes and conducted comparative analysis of *C. trogii* based on the obtained high-quality genomes. Rich genetic diversity was detected between the different strains of *C. trogii*. Moreover, significant differences between the two karyotypes included, but were not limited to, genome length, number of genes, and gene composition. Sequencing the genomes of these two karyotypes allowed us to gain a more comprehensive understanding of the genetic background of this strain.

*HSP20* genes represent the most abundant small heat shock protein (*sHSP*) genes in plants, and include, for example, 51 members in soybean (35), 48 in potato (14), and 41 in apple (15). Tandem and segmental duplications may be the major contributors of *HSP20* expansion. According to earlier work (18), fungi carry fewer *HSP20* genes, usually fewer than five. In this study, 14 *HSP20* members were identified in *C. trogii*, and this expansion may have been the result of tandem duplication. Most *HSP20* genes in plants have no or only one intron (14, 15, 21). According to previous studies, genes with few or no introns are considered to be rapidly activated in response to various stresses (36), and this is also the case for *HSP20* genes, as these genes usually have no intron and are more easily induced (14, 15). However, the *HSP20* genes in this study typically had two introns, indicating their differing evolutionary status from those from plants.

The induced high expression of *HSP20* genes can enhance thermotolerance in organisms (15, 17). However, in this study, the continuous high expression of *HSP20* genes at 35°C endowed *C. trogii* with high-temperature adaptive growth. Due to the high degree of sequence similarity, it was difficult to accurately analyze the expression levels of the duplicated genes. Benefiting from the genomic data of the different karyotypes, allele-specific expression analysis of *HSP20* genes was then conducted, and two duplicated allelic pairs (*Ct29HSP20.5*-*Ct31HSP20.5* and *Ct29HSP20.8*-*Ct31HSP20.8*) showed superior expression, indicating their indispensable role in high-temperature adaptive growth in *C. trogii*. The other two allelic pairs, *Ct29HSP20.6*-*Ct31HSP20.6* and *Ct29HSP20.7*-*Ct31HSP20.7*, which had low expression levels under all of the tested conditions, may be functionally redundant or may confer better fitness under other conditions (18). The responses of *HSP20* genes to heat stress may functionally contribute to the growth and survival of *C. trogii* at high temperatures, which might be useful for the selection and breeding of elite strains.

## MATERIALS AND METHODS

### Strains, cultivation, and fruiting body collection.

The dikaryotic *C. trogii* strain Ct001 was maintained on potato dextrose agar (PDA) at 4°C and stored at the Institute of Bioengineering, Guangdong Academy of Sciences. Fresh mycelial blocks (5 mm in diameter) were transferred to PDA plates kept at 25°C, 28°C, 32°C, 35°C, 37°C, or 42°C in the dark for 5 days. The growth rate was calculated based on hyphal radial growth under each condition. Monokaryotic strains with opposite mating types were obtained by methods previously reported (dedikaryotization via hyphal lysis, protoplast isolation, and colony regeneration) (37) from Ct001, and the monokaryons Ct001_29 and Ct001_31 were randomly selected.

The mycelia of Ct001 were cultured on minimal medium (MM) plates containing glucose (20 g/liter), (NH_4_)_2_SO_4_ (1.5 g/liter), K_2_HPO_4_ (1.0 g/liter), MgSO_4_ (0.3 g/liter), and vitamin B1 (0.5 mg/liter). Next, the glucose was replaced with an equal amount of four other carbon sources (20 g/liter), namely sucrose (SM), lignin (LM), cellulose (CM), and xylan (XM). Ct001 was then inoculated into the above four culture media, and the plates were further cultured at 25°C or 35°C for 5 days. For each sample (including mycelia cultured on PDA for 5 days at 25°C or 35°C), the mycelia were quickly scraped and mixed to produce a biological repeat sample, which was then frozen in liquid nitrogen and stored at −80°C. Three replicates were prepared for all of the treatments. The growth rates of Ct001 on different carbon sources were determined at 25°C and 35°C.

The 5-day-old mycelia of Ct001 cultured on PDA plates at 25°C were inoculated into culture vessels (300 ml) containing culture compost. The culture compost consisted of 10% oak wood, 70% sugarcane bagasse, 19% wheat bran, and 1% gypsum, with a final water content of 65%. The vessels were incubated at 25°C with approximately 50% humidity in the dark. These vessels were fully covered with mycelia after 18 days, at which point the surface of the medium was scraped with a sterilized scalpel and further cultivated for an additional 5 days. These vessels were then transferred to a fruiting room. The temperature was maintained at 28°C, and the room humidity was maintained at 85%, with a photoperiod of 12 h at 300 lx and 12 h in the dark. Three replicates of primordia (28 days after inoculation) and fruiting bodies (32 days after inoculation) were collected and quickly frozen in liquid nitrogen for further analysis.

### Genome and RNA sequencing.

Genome sequencing was performed using samples of Ct001_29 and Ct001_31. For each strain, 8 μg of genomic DNA extracted via the cetyltrimethylammonium bromide method (38) was conjugated to a 16-bp barcode sequence, and then a 20 kb-insert library was constructed. These libraries were sequenced in one single-molecule real-time (SMRT) cell on the PacBio sequel II platform. In addition, 10 μg of genomic DNA from both Ct001_29 and Ct001_31 was used to construct paired-end (PE) libraries with an average insert size of 300 bp. Sequencing of these libraries was performed on the NovaSeq platform (Illumina, Inc., USA).

RNA-Seq was performed on a total of 42 samples, including mycelia cultured on different media, at different temperatures, or with primordia or fruiting bodies. The total RNA extraction/quality control and sequencing library construction of all of the samples were conducted using methods previously reported (24). These libraries were sequenced on an Illumina NovaSeq platform, and paired-end 150-bp reads were generated.

### Assembly of the genome.

Raw read quality was assessed using FastQC (http://www.bioinformatics.babraham.ac.uk/projects/fastqc/), and low-quality bases or reads were filtered out using Skewer (39) with the following criteria: trimming from the 3′-end base to achieve a quality score of >30 and exclusion of reads with a length of <100 bp or an average quality of <30. The PacBio data were assembled using Canu v1.8 (40) and refined with Racon (41) and Pilon (42). *k*-mer analysis toolkit (KAT) (43) analysis and BUSCO analysis (44) with the fungi odb9 database were used to test the accuracy and completeness of our assembled genomes.

### Repeat sequence and gene annotation.

Dispersed repeated sequences at the DNA level were detected through an approach combining *de novo* prediction and homology-based searching. RepeatModeler v1.0.11 (http://www.repeatmasker.org/RepeatModeler/) was used to construct a *de novo* repeat library, and this *de novo* library was then mixed with Repbase (a database of eukaryotic repetitive elements) to conduct repeat searching using RepeatMasker v4.06 (http://www.repeatmasker.org/RMDownload.html).

Nuclear genomes for Ct001_29 and Ct001_31 were next annotated using EuGene v4.2 (45). Protein coding genes were functionally annotated by searching the following databases: Pfam (46), UniProt (https://sparql.uniprot.org/), and InterProScan (47).

### Gene family and phylogenetic analysis.

A total of 26 genomes (including Ct001_29, Ct001_31, and S0301 [30]) were used to conduct phylogenetic analysis, and the annotations and sequences of all of the other genomes considered were downloaded from NCBI (see Table S2 in the supplemental material). To identify gene family numbers, we performed an all-against-all comparison using BLASTP (48) with an E value cutoff of 1 × 10^−5^, and the OrthoMCL (49) method was used to cluster our BLASTP results into paralogous and orthologous clusters. The orthologous gene families calculated by OrthoMCL were subjected to CAFE5 (50) for identification of expansion and contraction using default parameters.

In total, 176 single-copy genes were used to construct a phylogenetic tree. Multiple-sequence alignments of these 176 genes were done using MUSCLE v3.8.31 (51); these are combined into a long sequence for each species. A phylogenetic tree was then constructed using RAxML v8.2.11 (52) with 1,000 bootstraps, the PROTGAMMAJTTF model, and Neurospora crassa and S. cerevisiae as the outgroups. Two divergence time calibration points were fixed in our molecule clock analysis. The most recent common ancestor (MRCA) of S. cerevisiae and *G. lucidum* diverged 723 Mya, and the MRCA of Ustilago maydis and *G. lucidum* diverged 466 Mya (timetree.org). The divergence times of the other nodes were then calculated using MCMCtree (53).

### Comparative genomics analysis.

The syntenic relationships among Ct001_29, Ct001_31, and S0301 (30) were analyzed using the MCScanX package (54) with default settings. Ct001_31 and S0301 were mapped to Ct001_29 using Minimap2 (55), and variants were called based on these mapping results. Variants with base changes shorter than 50 bp were defined as insertions/deletions (indels), and those longer than 50 bp were considered to be structural variations (SVs). When the number of overlapped bases (between genetic variations and genes) accounted for ≤0.2% of the total length of a gene, it was defined as a conserved gene, and when this ratio was ≥2%, this gene was defined as a variable gene. The predicted genes for Ct001_29 and Ct001_31 were then compared with each other, and the genes without any comparison results in the opposite genome were considered to be haplotype-specific genes.

For validation of the genetic variations and haplotype-specific genes between Ct001_29 and Ct001_31, mycelia from Ct001_29 and Ct001_31 were collected separately for DNA and RNA isolation after cultivation on PDA for 5 days at 25°C or 35°C. A total of 28 SNPs, 10 indels, and 8 SVs were randomly selected, and 20 primer pairs (several loci that could be amplified by 1 primer pair) were designed according to their conserved flanking sequences (see Table S3 in the supplemental material). PCR amplification was then conducted using genomic DNA for Ct001_29 and Ct001_31 as the templates. Three Ct001_29-specific genes and five Ct001_31-specific genes were randomly selected, and primer pairs were designed accordingly (Table S3). The cDNA from Ct001_29 and Ct001_31 was used as the amplification template. All of the primers were designed and synthesized by Tsingke Biotechnology Co., Ltd. (Beijing, China). All of the amplification products were analyzed using agarose gel electrophoresis or were sequenced at Tsingke Biotechnology Co., Ltd.

### Expression profile analysis.

The raw reads generated by RNA-Seq were trimmed and quality controlled using Skewer (39). HISAT and StringTie (56) were used to calculate the expression levels of each gene (transcripts per million [TPM]). DEGs between these different samples were identified using DESeq2 (57), and DEGs were defined as those genes that had a log_2_ fold change of ≥1 and a *P* value of ≤1E−3. To analyze the DEGs from mycelia grown on different carbon sources, pairwise comparisons of all of the expression data at 25°C were conducted, and the expression data at 35°C were processed similarly. The intersection of any pairwise comparison at 25°C and 35°C (15 groups in total; see Fig. S4A in the supplemental material) was considered to represent the DEGs responsive to carbon sources. To analyze the DEGs of mycelia grown at different temperatures, expression data between 25°C and 35°C on each carbon source were compared, and the intersection of all of these comparisons (six groups in total; see Fig. S4B) was considered to be those DEGs responsive to temperature. To analyze the DEGs during different developmental stages, the expression data of mycelia cultured at 25°C on PDA, primordia, and fruiting bodies were compared (Fig. S4C). An expression heatmap was drawn using pheatmap (https://cran.r-project.org/web/packages/pheatmap/index.html).

### Identification and analysis of the *C. trogii HSP20* gene family.

All of the annotated *HSP20* genes in Ct001_29 and Ct001_31 were manually corrected using Apollo (58) as previously reported (59), and genes with an HSP20 domain (Pfam identifier [ID] PF00011) were considered *HSP20* genes. Full-length protein sequences of two sets of *HSP20* gene members in Ct001_29 and Ct001_31 were used to construct a phylogenetic tree using RAxML (52). All of the *HSP20* genes from Ct001_29 were searched against those from Ct001_31 using BLASTP (48). By combining our phylogenetic tree and protein identity (≥85%), the allelic (one to one) relationship between these *HSP20* genes was confirmed. The duplication of *HSP20* genes was determined using the following two criteria: (i) the protein length of the shorter sequence covered ≥50% of the longer sequence, and (ii) the similarity of the two aligned sequences was ≥90%.

The expression of allelic genes was calculated as the average sequencing depth of SNP loci between allelic genes and was normalized by the total read count of three replicates. The expression level of duplicated *HSP20* genes was verified based on clone sequencing. Amplification and cloning of partial sequences (563 bp) of *HSP20.5* to *HSP20.8* was then conducted. A primer pair (5′-CCCCCTTTCTCCCTCACTA-3′ and 5′-AACMACAACCATCTCCWCCRT-3′) for these genes was designed, and PCR was conducted based on the cDNA obtained from mycelia cultured on PDA at 35°C. The amplification products were cloned, and 56 clones were sequenced at Tsingke Biotechnology Co., Ltd.

The synthesized cDNA for the RNA-Seq was used for qPCR. ChamQ Universal SYBR qPCR master mix (Vazyme, Nanjing, China) was used for qPCR as described previously (24). Glyceraldehyde 3-phosphate dehydrogenase (*GAPDH*) was used as a reference. Primers (see Table S3 in the supplemental material) were designed and synthesized by Tsingke Biotechnology Co., Ltd.

### Gene functional enrichment analysis.

Gene ontology (GO) enrichment analysis was carried out on genes in expanded families, conserved/variable genes, haplotype-specific genes, and DEGs using clusterProfiler (60), and enrichment results with a *P* value of <1 × 10^−3^ were retained.

### Data availability.

The genome sequences and raw sequencing data have been deposited in GenBank under project accession number PRJNA747173 and PRJNA749681. All of the sequences and related annotations, including nuclear genomes and RNA-Seq data, can be accessed from the Global Pharmacopoeia Genome Database (GPGD) (61) at the following URL: http://www.gpgenome.com:8080/species/62343.
