# Supplementary material for: Haploid Genome Analysis Reveals a Tandem Cluster of Four HSP20 Genes Involved in the High-Temperature Adaptation of Coriolopsis trogii
Source: Microbiol Spectr. 2021 Aug 18;9(1):10.1128/spectrum.00287-21. doi: 10.1128/spectrum.00287-21 (PMC8552761; doi:10.1128/spectrum.00287-21)
Supplement: SUPPLEMENTAL FILE 1 — Supplemental material. Download SPECTRUM00287-21_Supp_1_seq9.pdf, PDF file, 1.5 MB [file spectrum00287-21_supp_1_seq9.pdf]

TABLE S1 Statistics of the repeat elements of the Ct001_29 and Ct001_31 genomes

Type			Ct001_29			Ct001_31		
			#elements	length(bp)	%genome	#elements	length(bp)	%genome
Retroelements			649	909,636	2.34	811	1,513,157	3.76
	LINEs:		-	-	0.00	70	124,967	0.31
		L2/CR1/Rex	-	-	0.00	70	124,967	0.31
		RTE/Bov-B						
		L1/CIN4						
	LTR elements:		649	909,636	**2.34**	741	1,388,190	**3.45**
		BEL/Pao	42	58,844	0.15	33	3,207	0.01
		Ty1/Copia	266	376,367	0.97	272	674,221	1.68
		Gypsy/DIRS1	320	448,124	1.15	417	681,180	1.69
DNA transposons			152	149,229	0.38	136	113,507	0.28
	hobo-Activator		112	124,663	0.32	23	35,337	0.09
	Tc1-IS630-Pogo		22	14,139	0.04	24	24,455	0.06
	PiggyBac		-	-	0.00	13	1,896	0.00
	Tourist/Harbinger		-	-	0.00	17,529	7,222,040	0.42
Rolling-circles			67	98,206	0.25	47	103,329	0.26
Unclassified:			3,159	1,611,718	4.15	3,805	1,987,337	4.94
Total interspersed repeats:				2,670,583	6.87		3,614,001	8.99
Small RNA:			38	54,509	0.14	25	99,372	0.25
Satellites:			-	-	0.00	-	-	0.00
Simple repeats:			5,124	229,510	0.59	5,203	234,043	0.58
Low complexity:			827	42,808	0.11	805	42,274	0.11

TABLE S2 Species used in the phylogenetic analysis

Abbreviation	Species	Source
Asub	*Auricularia subglabra*	GCA_000265015.1
Clac	*Cystobasidiopsis lactophilus*	GCA_001599975.1
Dsqu	*Dichomitus squalens*	GCA_000275845.1
Fpin	*Fomitopsis pinicola*	GCA_000344655.2
Gluc	*Ganoderma lucidum*	GCA_000271565.1
Gsin	*Ganoderma sinense*	GCA_002760635.1
Ncra	*Neurospora crassa*	GCA_000182925.2
Pcar	*Phanerochaete carnosa*	GCA_000300595.1
Post	*Pleurotus ostreatus*	GCA_003313235.2
Ppla	*Postia placenta*	GCA_002117355.1
Pstr	*Puccinia striiformis*	GCA_011750755.1
Scer	*Saccharomyces cerevisiae*	GCF_000146045.2
Tcin	*Trametes cinnabarina*	GCA_000765035.1
Tcoc	*Trametes coccinea*	GCA_002092935.1
Thir	*Trametes hirsuta*	GCA_001302255.2
Tpol	*Trametes polyzona*	GCA_001939255.1
Tpub	*Trametes pubescens*	GCA_001895945.1
Tsan	*Trametes sanguinea*	GCA_008973685.1
Tspx	*Trametes sp. AH28-2*	GCA_001304625.1
Ct001_29	*Coriolopsis trogii*	this study
Ct001_31	*Coriolopsis trogii*	this study
S0301	*Coriolopsis trogii*	GCA_007896425.1
Tver	*Trametes versicolor*	GCA_000271585.1
Tvil	*Trametes villosa*	GCA_002964805.1
Umay	*Ustilago maydis*	GCA_000328475.2
Wcoc	*Wolfiporia cocos*	GCA_000344635.1

TABLE S3 Primers used in the validation of genetic variations, haplotype-specific genes and expression quantification of *HSP20* tandem genes

Primer_name	Forward (5'-3')	Reverse (5'-3')	SNP loci	Indel loci	SV loci	Haplotype-specific	Length
SNP_1	ACCTCCAGTCTCCATCACAAC	CCTCTCGTCGAGCCTATGTT	1	-	-	-	-
SNP_2	AAGGCAATGGTATCAAGACT	CACAGACAGGAACGAACA	11	-	-	-	-
SNP_3	CGACGAGGATGAGGAAGA	TAGTTGAGACCGAGTAGGAT	1	-	-	-	-
SNP_4	ATCGTTGGCGTTGGTGAGT	CGGTGAGCGTAGGAATGAGT	2	-	-	-	-
SNP_5	TACGCTGTCATCACTGTC	GACCGAAGAGGAAGTAGAC	3	-	-	-	-
Indel_1	GTAGTGTTCGTTGCTGTTG	CTCATCGCTGCTCATCTC	-	1	-	-	-
Indel_2	GCAACAGATGAGCAGTGA	AGGAGAAGAGGCAGGATG	-	2	-	-	-
Indel_3	AAGAGGAGGAGGACGATAG	TGTCAGAGGTAGGAGTGTT	3	1	-	-	-
Indel_4	GAGCAAGAAGGCGAAGAA	TGAAGTAGAGCACGAACAG	2	2	-	-	-
Indel_5	CGACCGTGGAGGACTTCAA	GACAGGACAGGACAGGAGAC	-	Sequencing failed	-	-	-
Indel_6	GACTTCCTACGCTATATGTTCAACT	CAGAGGTCGCTTGGTTCACT	2	1	-	-	-
Indel_7	CAGCAACCAGACAGACAT	GGACATTCACTCACTCACT	1	2	-	-	-
SV_1	CCAATCCAGTCTCCAGTATC	CCTTCACCGTAGTCATTCTT	-	-	1	-	-
SV_2	TTGGACTCTGCTGGCTAA	CTGTATTCGTGCGTTGGA	-	-	1	-	-
SV_3	GTCCTCCGATATTCCTTCC	TTGGCGTGTTGTTGGTAT	-	-	1	-	-
SV_4	CGCATTCTCTATATTGTCCTTC	GGTAAGTCGTAGCATCATTATC	-	-	1	-	-
SV_5	CGACGAGCATAGCATCAG	CCGACCTACGACAGTTCT	-	-	1	-	-
SV_6	GGAATACCAGCAAGACCAT	GAGACAGATACGAGAGGAAG	-	-	1	-	-
SV_7	GGTATCGGAGCCATTGAAT	GTTATCTGGTAGTGTTGTAGTC	-	-	1	-	-
SV_8	CGTATGGCAAGTCAACCT	GTCCTCTGATTCGCTCTG	-	-	1	-	-
Ct31g0128441	GACTGCGACCACATACAA	CGAGATAGTGCTTCCAACA	-	-	-	Ct001_31	-
Ct31g0128431	ATGTTGGAGGCTGTGAAG	TACGAATAGTCTGTGCTTGA	-	-	-	Ct001_31	-
Ct31g0076031	GCATACCTATCCGCAGAC	CGAGTGAAGATGACAGTGT	-	-	-	Ct001_31	-
Ct31g0069651	GACAAGCGGAGGATGATG	TGAAGAAGATATAGGACGAGAC	-	-	-	Ct001_31	-
Ct31g0038071	ATGGTCATCCTCTGTGGTA	CGATTCTTGTCTGGTCTCA	-	-	-	Ct001_31	-
Ct29g0129561	CTCAAGTCCATCGCCTAC	TCTCCACCAAGCACAATC	-	-	-	Ct001_29	-
Ct29g0090471	TCCGTATGAGAGTGCTATTG	GGTGGTCTTGTCGTTCTG	-	-	-	Ct001_29	-
Ct29g0008441	CTGGAACCGTCGTAACAA	TAGAGCACTGGAGATGGAA	-	-	-	Ct001_29	-
GAPDH	CCGTTGGTGCTGATTACA	CGTTGGAGATGACTTGGTA	-	-	-	-	183
hsp20.5<br>hsp20.6<br>hsp20.7<br>hsp20.8	TCTTCACCGAGCCGTTCTAC	CCGTTACGAGGTTCTTGTCTTG	-	-	-	-	180

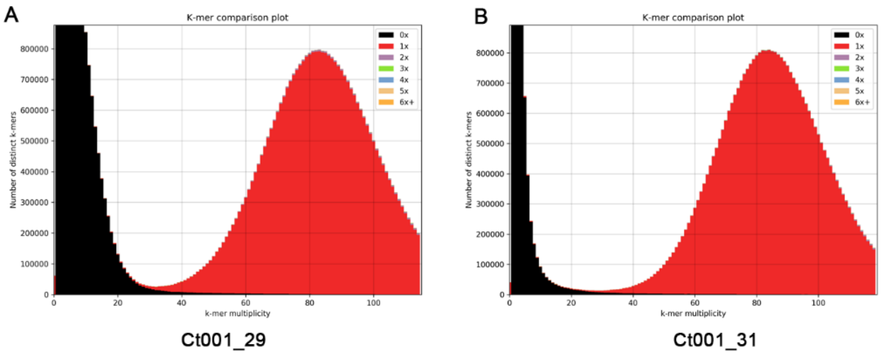

FIG S1 *K*-mer spectrum analysis. Comparison of 27-mer between assembled contigs and long reads by the KAT program. 0x – 6x+ means *k*-mers of long reads could be found in contigs *k*-mers 0 – 6+ times. There was no peak of 0x indicating no fragment loss in the assembly.

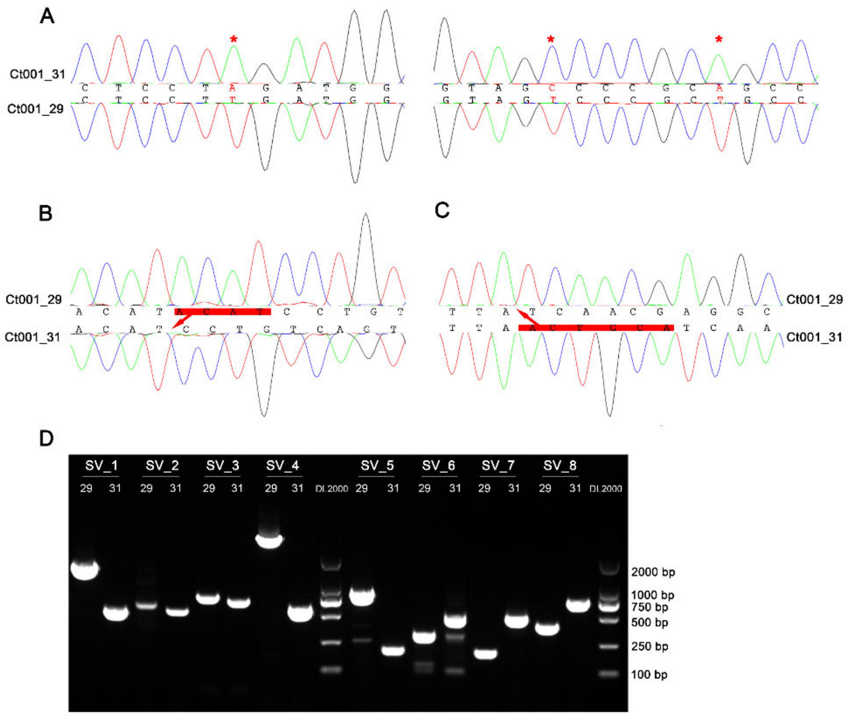

FIG S2 Validation of genetic variations between Ct001_29 and Ct001_31. (A) Single nucleotide polymorphisms, red asterisks represent nucleotides with differences. (B) and (C) Insertions/Deletions, nucleotides in red rectangles represent insertions. (D) Structural variations, 29 and 31 represent Ct001_29 and Ct001_31, respectively.

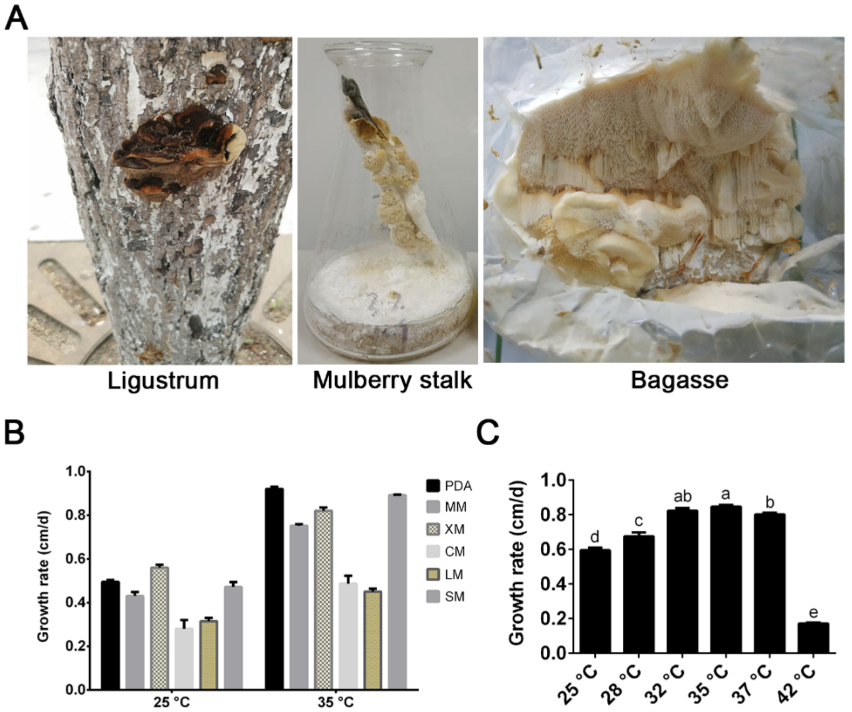

FIG S3 Fruiting bodies and mycelial growth rate of the *C. trogii* strain Ct001. (A) Fruiting bodies of *C. trogii* produced on natural substrates. (B) Growth rate of mycelia cultured on different carbon sources and under different temperatures. (C) Growth rate of *C. trogii* cultured on PDA at different temperatures, different lowercase letters represent significant differences ($P < 0.05$).

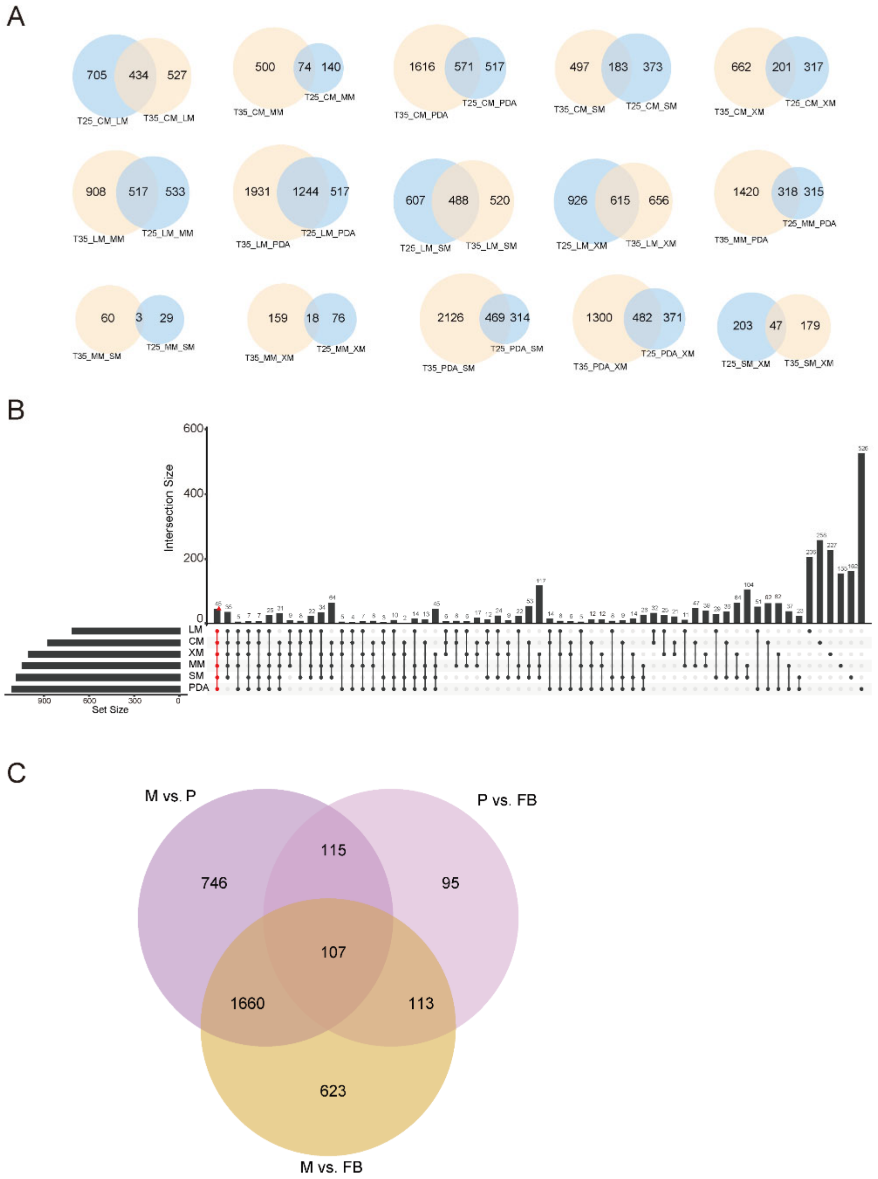

FIG S4 Identification of differentially expressed genes (DEGs). (A) Venn diagram of carbon-related DEGs. 25°C (T25), 35°C (T35), glucose (MM), sucrose (SM), lignin (LM), cellulose (CM), and xylan (XM). A total of 2,243 DEGs were identified. (B) DEGs detected between 25°C and 35°C on different carbon sources. A total of 155 DEGs were identified (under any combination of five different carbon groups). (C) Venn diagram of DEGs of developmental stages. A total of 2,836 DEGs were identified.

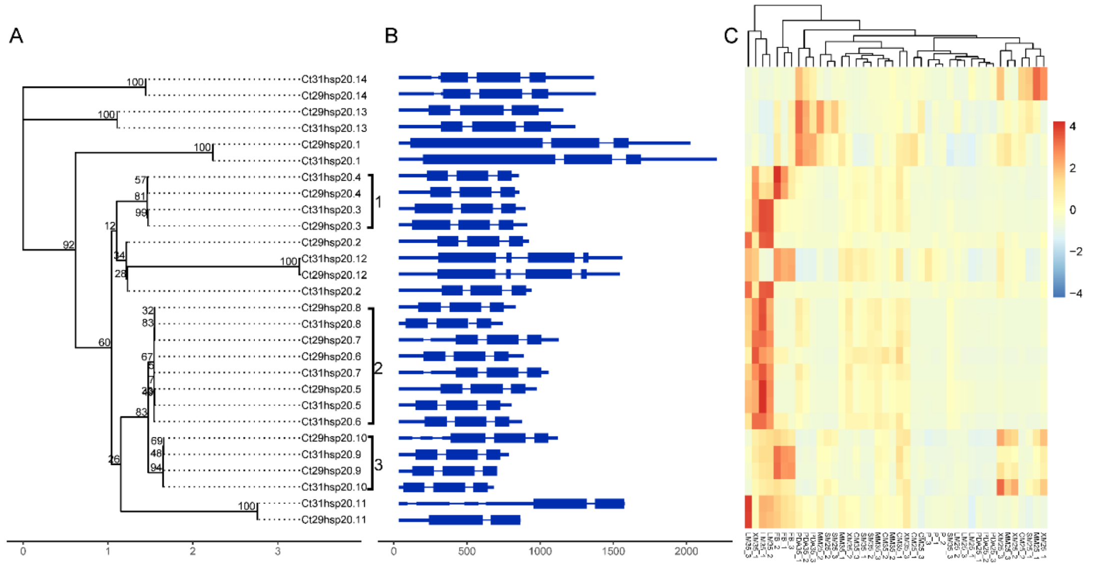

FIG S5 Phylogenetic relationship (A), gene structure (B), and expression pattern (C) of *HSP20*s of *C. trogii*. Numbers on the tree represent supporting values of 1000 bootstraps, and numbers 1–3 behind gene names represent three groups of duplicated genes. Blue rectangles represent CDSs, thin blue lines connecting two exons represent introns, thick blue lines represent the 5’-UTR or 3’-UTR. Glucose (MM), sucrose (SM), lignin (LM), cellulose (CM), xylan (XM), potato dextrose agar (PDA), primordium (P), and fruiting body (FB).

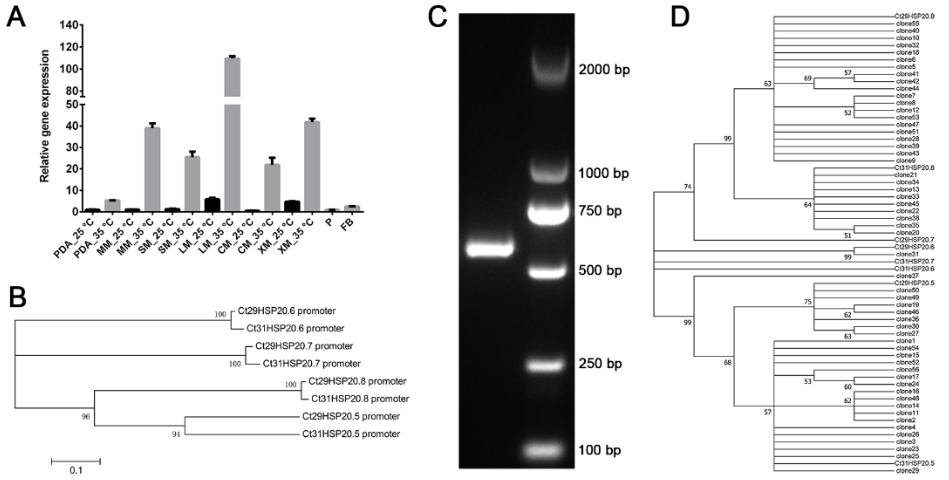

FIG S6 Expression profiles and promoter features of the *HSP20* tandem cluster. (A) Gene expression detected by qPCR, glucose (MM), sucrose (SM), lignin (LM), cellulose (CM), xylan (XM), potato dextrose agar (PDA), primordium (P), and fruiting

body (FB). (B) Promoter sequence similarity analysis. Numbers on phylogenetic tree represent supporting values of 1000 bootstraps. (C) Agarose gel electrophoresis of a shared 563 bp region of *HSP20* tandem genes. (D) A neighbor-joining tree showing relationships among the PCR product (563 bp)-derived 56 clones and *HSP20* tandem genes. Numbers on the NJ tree represent supporting values of 1000 bootstraps. Clades with support of less than 50 were cut.
